# Genes Found Essential in Other Mycoplasmas Are Dispensable in *Mycoplasma bovis*

**DOI:** 10.1371/journal.pone.0097100

**Published:** 2014-06-04

**Authors:** Shukriti Sharma, Philip F. Markham, Glenn F. Browning

**Affiliations:** Asia-Pacific Centre for Animal Health, School of Veterinary Science, The University of Melbourne, Parkville, Victoria, Australia; University of Florida, United States of America

## Abstract

Mycoplasmas are regarded to be useful models for studying the minimum genetic complement required for independent survival of an organism. *Mycoplasma bovis* is a globally distributed pathogen causing pneumonia, mastitis, arthritis, otitis media and reproductive tract disease, and genome sequences of three strains, the type strain PG45 and two strains isolated in China, have been published. In this study, several Tn*4001* based transposon constructs were generated and used to create a *M. bovis* PG45 insertional mutant library. Direct genome sequencing of 319 independent insertions detected disruptions in 129 genes in *M. bovis*, 48 of which had homologues in *Mycoplasma mycoides* subspecies *mycoides* SC and 99 of which had homologues in *Mycoplasma agalactiae*. Sixteen genes found to be essential in previous studies on other mycoplasma species were found to be dispensable. Five of these genes have previously been predicted to be part of the core set of 153 essential genes in mycoplasmas. Thus this study has extended the list of non-essential genes of mycoplasmas from that previously generated by studies in other species.

## Introduction

Mycoplasmas are a group of obligately parasitic bacteria that evolved from Gram positive organisms by reductive evolution. In the process, they have lost many dispensable genes and are thought to maintain only regulatory systems essential for their survival *in vivo* [1]–[6].

The mycoplasmas lack a cell wall and have relatively small genomes (580 to 1380 kbp), but can still perform all the functions required for autonomous life [4], [5]. Despite their genetic simplicity, many are pathogenic and can persist for very extended periods in their vertebrate hosts. *Mycoplasma bovis*, a significant pathogen of cattle throughout the world, lies in the hominis phylogenic group, with *M. agalactiae*, *M. fermentans*, *M. synoviae*, *M. pulmonis*, *M. hyopneumoniae*, *M. arthritidis*, *M. hominis*, *M. conjunctivae*, *M. crocodyli*, *M. mobile* and *M. orale* [7], [8].

The genomes of three strains of *M. bovis*, the type strain PG45 [9] and two strains isolated in China, Hubei-1 [10] and HB0801 [11], have been determined. There have been very few functional studies on *M. bovis*, and its virulence factors and the mechanisms involved in its pathogenicity are largely unknown. However, it is clear that it uses complex strategies to invade and avoid the immune response of the host [12], [13].

Only a few tools are available to genetically manipulate mycoplasmas. Transposons have been used to disrupt genes to study their role in virulence and their immunogenicity, to define the minimum genetic complement required for independent survival of an organism [14]–[17], and as vectors for xenogeneic expression [18], [19]. Only Tn*916* and Tn*4001*, isolated from *Enterococcus faecalis* and *Staphylococcus aureus*, respectively, have been shown to function in mycoplasmas. Tn*4001* is smaller (4.7 kbp) than Tn*916* (18 kbp) and appears to have a better transformation efficiency [20], and plasmid pISM*2062*, carrying the transposon Tn*4001* [21], has been used to introduce this transposon into *M. bovis* [22].

In the study described here, a library of *M. bovis* strain PG45 mutants was created by transformation with Tn*4001*-based plasmids. The locations of transposon insertions in the genome were identified by genomic sequencing and the catalogue of disruptable genes compared to those generated in other pathogenic mycoplasmas to identify those genes previously thought to be indispensible in mycoplasmas that are dispensable in *M. bovis*.

## Results

### Functionality of transposon constructs for *M. bovis* strain PG45

The series of constructs based on Tn*4001* were initially examined for their ability to transform *M. gallisepticum* strain S6, which was considered a model organism for transformation, as it had been transformed successfully in previous studies in our laboratory [23], [25], [27]. Following success in transforming *M. gallisepticum*, pTn*4001*complete was used to transform *M. bovis* strain PG45. Subsequently, *M. bovis* was transformed with pTn*4001*single and then with the minitransposons containing either the gentamicin or tetracycline resistance genes. Individual colonies on selective agar plates were selected and cultured in appropriate selective broth and the cultures examined by PCR to confirm the presence of the gentamicin or tetracycline resistance genes.

### Randomness of transposon integration

**Figure 1:**
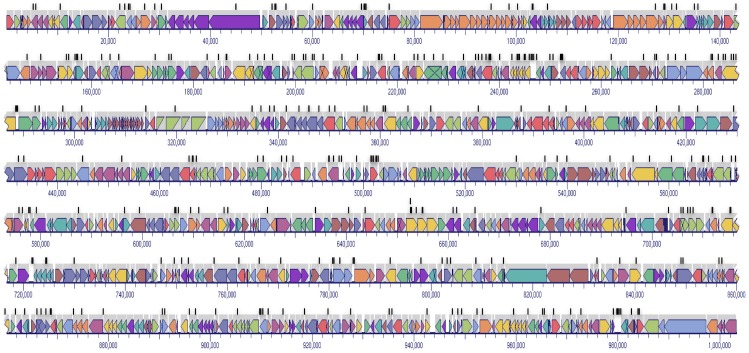
Location of 319 transposon integration sites in the *M. bovis* genome. The distribution of the transposon insertion sites indicates that insertions were randomly distributed.

The randomness of transposon integration in the genome was confirmed by direct genomic sequencing of the mutant library (Figure 1), which allowed mapping of the transposon integration site for 319 mutants.

### Haystack screening for a *xer1* insertion

To identify mutants with a disruption in a specific gene target, transposon insertion sites were initially screened using a PCR-based strategy based on the haystack mutagenesis approach. For each of the four targeted loci, a first round of PCR was performed on each pool using one primer specific for the GOI and a second that would bind to the 5′ or 3′ end of Tn4001. An amplification product was identified in pool 5 using the oligonucleotide primer pair GKxer1 for and IR inverse (Table S1), indicative of a *xer1* disruption. The pool contained 29 individual mutants. The second round of PCR was performed on DNA from each the 29 mutants within the pool individually and mutant number 29, which had the *xer1* gene disrupted by Tn4001complete, was identified. The PCR yielded an amplification product of around 350 bp, suggesting that the site of insertion of the transposon was expected to be around 350 bp downstream of the start codon of the gene. This was confirmed by cloning the PCR product in pGEM-T and sequencing the insert. Haystack screening did not detect disruptions in p48, oppD or the restriction endonuclease gene, and the absence of these mutations from the library was confirmed by direct genome sequencing.

### Non-essential genes in *M. bovis*

After initial studies using haystack mutagenesis, we used direct sequencing to identify the insertion sites in all the mutants in the library. Of the 319 mutants, 151 were generated using pTn*4001*single, 125 using pTn*4001*complete, 40 using pMiniTn*4001*-gent and 3 using pMiniTn*4001*-tet. A total of 191 insertions were in annotated ORFs, 38 within predicted intergenic regions, 40 within ICE elements and 50 within transposase genes. Of the 191 insertions in ORFs, 113 were in predicted genes, 56 in genes encoding membrane proteins or lipoproteins and 22 in genes encoding hypothetical proteins. Based upon the criteria for gene disruption, 129 genes had been disrupted, and of these 48 and 99 genes had homologues in *M. mycoides* subspecies *mycoides* SC strain PG1 and *M. agalactiae* strain PG2, respectively (Table 1). There were 21 additional genes that had transposon insertions within the last 15% of the coding sequence and which were therefore not considered to be disrupted, although this may not have been the case if function was located in this region of the protein (Table S2). Several genes were disrupted in multiple mutants. Intergenic regions contain promoters for genes located downstream, so transposon insertions in intergenic regions may have impaired the function of downstream genes or operons, so while these insertion events were not considered gene disruptions, the mutants carrying them (Table S3) may also be important in assessment of gene function. In addition, a total of 90 insertions were observed within integrative conjugative elements (ICE) (Table S4) and transposase genes (Table S5).

**Table 1:** Non-essential genes in *M. bovis* strain PG45 identified by transposon mutagenesis and their homologues in *M. mycoides* subspecies *mycoides* SC and *M. agalactiae*.

ORF	Product	Gene	Gene locus	Gene size (bp)	Tn insertion site in gene (%)	MSC orthologue*	MAGPG2 orthologue*
0008	Oxidoreductase, FAD/FMN-binding protein		5995–4814	1182	14.5& 86.2	Y	Y
0024	S41B peptidase family lipoprotein		23202–21331	1872	48.2		
0025	Indigoidine synthase A family protein		24378–23473	906	30.6 &45.8		Y
0028	Membrane protein		26308–28284	1977	39.1		Y
0030	Hypothetical protein		29413–29760	348	40.5		Y
0031	Hypothetical protein		29735–31156	1233	70.6		
0038	Hypothetical protein		49660–39680	9980	49.3		Y
0049	Membrane protein		59330–60364	1035	80.8		Y
0060	tRNA modification GTPase	*trmE*	69942–68605	1338	45.1	Y	Y
0061	Hypothetical protein		70070–70744	675	19.3, 20.3 &74.8		Y
0065	50S ribosomal protein L34	*rpmH*	74593–74742	150	7.3	Y	Y
0083	DNA polymerase IV		94778–96025	1248	44.1	Y	Y
0086	Lipoprotein		97862–99055	1194	33.6		Y
0093	Membrane protein		104021–102738	1284	35.0 & 37.1		Y
0096	RNA methyltransferase, TrmH family		105512–106243	732	78.1	Y	Y
0116	Oligopeptide ABC transporter ATP-binding protein		125479–127881	2403	72.0		Y
0118	Membrane protein		128948–129439	491	1.0 & 42.2		Y
0119	Membrane protein		129526–130743	1218	80.9		
0120	Lipoprotein		130781–132721	1941	28.8		
0123	Membrane protein		135436–135822	387	36.2	Y	Y
0131	LemA family protein		142685–143383	699	80.8		Y
0133	Oligoendopeptidase F	*pepF*	147853–146012	1853	2.7	Y	Y
0135	Polyamine ABC transporter permease	*potB*	149512–150348	837	80.8	Y	Y
0139	Membrane protein		153571–155238	1667	81.0		Y
0140	Thiamine biosynthesis protein/tRNA modification protein	*thiI*	155240–156376	1137	34.7	Y	Y
0153	Phosphate acetyltransferase	*pta_1*	171898–172854	957	60.4		Y
0157	Membrane protein		175094–176596	1503	1.5 & 49.3		Y
0164	Lipase/esterase LIP3/BchO family		185490–184690	801	48.4	Y	Y
0168	TypeIII RM system methylase		191805–190104	1702	45.4		
0169	Type III RM system methylase		193542–191868	1674	38.7		
0170	Type III RM system methylase		195267–193599	1668	80.2		
0176	Membrane protein		203450–201144	2307	0.9, 58.1 & 71.6		
0215	Membrane nuclease A	*mnuA*	250107–248878	1329	2.3		Y
0216	Peptidase, M17 family		250218–251579	1362	38.0		Y
0227	Methionine adenosyltransferase	*metK*	261973–263121	1149	1.5	Y	Y
0232	Membrane protein		267520–269451	1932	53.5	Y	Y
0234	Lipoprotein		270776–270095	624	45.8		Y
0237	Type I RM system methylase	*hsdM-1*	272909–275587	2679	18.0 & 52.7		Y
0238	Type I RM system, S subunit		275592–276749	1158	79.5		Y
0241	Type I RM system, S subunit		282015–280936	1194	17.2 & 58.2		Y
0243	Hypothetical protein		288402–283690	4712	36.4, 48.9, 60.2, 82.1 & 99.1		
0245	Hypothetical protein		291827–293329	1503	52.8 & 95.0		Y
0281	HAD-superfamily hydrolase		313881–314750	869	12.9		
0957	23S ribosomal RNA	*rrl_3*	317923–320807	2884	52.7		
0298	Lipoprotein		335476–334424	1053	44.4		Y
0300	Deoxyribose-phosphate aldolase	*deoC*	337090–336422				
	669	75.5	Y	Y			
0301	Pyrimidine-nucleoside phosphorylase	*pdp*	338395–337100	1296	2.1	Y	Y
0307	Oligosaccharide ABC transporter permease		344498–343515	983	39.3		Y
0309	Oligosaccharide ABC transporter ATP-binding protein		347590–345488	2103	75.8 & 76.6	Y	Y
0310	Lipoprotein, nuclease family		348768–347599	1170	70.3		Y
0311	Membrane lipoprotein P81	*mb-mp81*	351020–348834	2187	44.1		Y
0316	Membrane protein		357175–355469	1707	35.2		Y
0317	Deoxyribonuclease IV phage-T4-induced	*nfo*	358043–357207	837	70.5	Y	Y
0327	AEC family transporter		369686–370930	1245	18.2	Y	Y
0333	Kinase family protein		379206–377755	1452	12.5		Y
0349	Membrane protein		396678–395692	987	25.5		Y
0353	Lipoprotein		401201–400053	1149	64.7	Y	Y
0366	Type II restriction enzyme		414369–413662	708	10.9		
0370	Membrane protein		419556–418309	1248	65.9		Y
0372	Membrane protein		421848–424058	2211	29.9		Y
0375	Membrane protein		429203–431452	2250	14.0		Y
0376	LppD family lipoprotein		431482–434052	2571	38.5 & 73.1		Y
0385	Lipoprotein		446177–443793	2385	43.8	Y	Y
0390	Glycosyltransferase		452829–451825	1005	24.7		Y
0402	Lipoprotein		465914–463971	1944	9.2	Y	Y
0403	msrA/msrB peptide methionine sulfoxide reductase		466880–465951	930	23.8 & 58.2	Y	Y
0404	Smr domain-containing protein		467245–466970	276	6.9		Y
0416	Lipoprotein		479242–481113	1872	65.9		Y
0419	Hypothetical protein		483086–484111				
	1025	82.8	Y	Y			
0421	S41B peptidase family lipoprotein		487636–485678	1959	5.17		
0425	Membrane protein		495066–493078	1989	46.0, 88.2 & 93.4		
0458	Hypothetical protein		530597–529914	684	76.2		Y
0464	Potassium transporter, Trk family		535963–535295	669	26.3	Y	Y
0466	Membrane protein		537715–538167	453	62.9		Y
0468	Hypothetical protein		539519–540037	519	53.2		
0503	Lipoprotein		580958–578787	2172	78.9	Y	Y
0509	Membrane protein		587125–588648	1524	31.5	Y	Y
0519	ABC transporter permease protein		596244–597584	1340	37.5		Y
0520	ABC transporter ATP-binding protein/chromosome segregation protein	*smc*	600615–597637	2979	38.6	Y	Y
0527	DHH family protein		606297–607298	1002	11.3, 20.4 & 64.5		Y
0529	Glycerol kinase	*glpK*	609112–610620	1509	22.3	Y	Y
0530	Glycerol uptake facilitator protein	*glpF*	610629–611420	792	51.9	Y	Y
0533	Neutral amino acid transporter, L-type amino acid transporter LAT family		614828–616498	1671	65.8	Y	Y
0534	Hypothetical protein		616549–617004	456	29.6	Y	Y
0550	Membrane protein		633844–635634	1791	11.5		
0557	S41B peptidase family lipoprotein		643754–645631	1877	4.1		Y
0564	Lipoprotein		651735–654044	2310	39.6, 39.6, 46.5 & 92.2		Y
0565	Lipoprotein		654047–655885	1839	43.8 & 47.9		
0568	DAACS family amino acid transporter		661162–659552	1611	20.9	Y	Y
0569	Pyridine nucleotide-disulfide oxidoreductase		662504–661155	1350	55.5	Y	Y
0584	Lipoprotein		677916–678983	1068	52.2		Y
0617	Type I RM system R subunit	*hsdR*	705566–707224	1659	16.4, 36.1 & 75.3		
0618	Type I RM system M subunit	*hsdM-2*	707238–708689	1452	16.3 & 67.9		
0621	Type I RM system S subunit	*hsdS*	712712–711396	1272	49.3 & 49.6		
0623	Hypothetical protein		715793–713523	2271	4.3 & 4.7	Y	
0629	Non-specific serine/threonine protein kinase	*pknB*	724478–723480	999	23.3		Y
0655	Lipoprotein		746841–747865	1024	27.6		
0662	Hypothetical protein		752431–752135	297	31.3	Y	
0669	CvpA family protein		763380–761983	1398	80.8		Y
0673	Membrane protein		767508–765733	1776	62.6		
0685	Membrane protein		780789–783107	2319	11.1 & 57.7		
0690	Ser/thr protein phosphatase family/5′-nucleotidase, C-terminal domain-containing protein		793088–791046	2043	54.9		Y
0695	Drug resistance ATPase ABC transporter family, ATP-binding protein		797866–799479	1614	17.0	Y	Y
0719	S1 RNA binding domain-containing protein		836712–838871	2160	18.7	Y	Y
0720	ATP-dependent chaperone protein	*clpB*	839018–841186	2169	62.0		Y
0728	Phosphoglucomutase/phosphomannomutase domain-containing protein		848314–851286	2973	35.4 & 47.1		Y
0734	Orotidine 5′-phosphate decarboxylase	*pyrF*	856735–856082	654	61.9		Y
0735	Phosphoenolpyruvate-dependent sugar phosphotransferase system, EIIA 2		857233–856745	489	9.8	Y	Y
0738	Phosphotriesterase family protein		860537–859476	1062	18.6		Y
0743	Type III RM system		865850–867709	1859	16.5 & 68.9		
0744	N-6 adenine-specific DNA methylase truncated		867719–868585	867	17.4		
0748	Glycerol ABC transporter permease	*gtsC2*	873847–873035	813	74.2	Y	Y
0749	Glycerol ABC transporter permease	*gtsB2*	874811–873837	975	25.3	Y	Y
0766	Site-specific DNA-methyltransferase adenine-specific		890583–891707	1125	1.2 & 42.4		Y
0770	Ribonuclease HIII	*rnhB-2*	896491–895868	624	68.8	Y	Y
0777	Hypothetical protein		901078–901689	612	12.6		Y
0787	Membrane protein		911272–911937	666	17.0	Y	
0800	Hypothetical protein		923232–922786	447	10.5	Y	Y
0810	Variable surface lipoprotein G	*vspG*	935674–934715	960	28.2		
0822	Site-specific recombinase, phage integrase	*xer1*	948412–949161	750	45.9		Y
0825	Lipoprotein		951540–952691	1152	84.5		Y
0826	Hypothetical protein		952881–955298	2418	22.1		Y
0831	Ribosomal large subunit pseudouridine synthase, RluA family		959814–958960	855	3.4	Y	Y
0838	Hypothetical protein		965052–964435	617	6.7	Y	
0839	Chaperone protein	*dnaJ*	966317–965184	1134	61.0	Y	Y
0845	tRNA binding domain-containing protein		970893–970291	603	7.1	Y	Y
0849	Methyltransferase, HemK family		973014–972292	723	50.3	Y	Y
0855	SsrA-binding protein	*smpB*	978922–979368	447	31.5	Y	Y
0858	Transcriptional regulator		983085–982126	960	25.8		Y

*M. bovis strain PG45 has 382 genes homologous to those of *M. mycoides* subspecies *mycoides* SC strain PG1 (MSC) and 595 homologous to those of *M. agalactiae* strain PG2 (MAGPG2); Y indicates the presence of an orthologous gene.

A number of notable genes were disrupted, including those annotated as encoding the heat shock proteins ClpB (MBOVPG45_0720) and DnaJ (MBOVPG45_0839), all the genes in the putative nucleotide transporter operon (MBOVPG45_307 to MBOVPG45_311), one gene in the polyamine ABC transporter system operon (MBOVPG45_0135), two genes in the glycerol ABC transporter system operon (MBOVPG45_0748 & MBOVPG45_0749), and in the genes encoding the glycerol kinase (MBOVPG45_0529) and the glycerol uptake facilitator protein (MBOVPG45_0530).

### Fewer essential genes in mycoplasmas than in previous studies

In an early study employing transposon mutagenesis, 310 genes were reported to be essential in *M. pulmonis* [15]. A further study on *M. pulmonis* found an additional 39 of these 310 genes to be dispensable [14], and it has been suggested that there are 153 core essential genes in *Mycoplasma* species [26]. In the study described here on *M. bovis*, 23 genes considered to be essential in *M. pulmonis* in the initial study [15], 16 of which were still found to be essential in the subsequent study [14], were disrupted (Table 2). Five of these genes, encoding the tRNA modification GTPase TrmE (MBOVPG45_0060), the polyamine ABC transporter permease PotB (MBOVPG45_0135), the methionine adenosyltransferase MetK (MBOVPG45_0227), the chaperone protein DnaJ (MBOVPG45_0839) and the *ssr*A binding protein SmpB (MBOVPG45_0855), were considered essential in all previous gene essentiality studies in mycoplasmas [14]–[16], [28] and have been predicted to form the core set of 153 essential genes in mycoplasmas [26]. Thus our study has demonstrated that mycoplasmas have fewer core essential genes than predicted previously.

**Table 2:** Genes regarded as essential in previous studies found to be dispensable in this study.

ORF	Product	Gene	Gene size (bp)	Essential MYPU orthologue^1^ (% amino acid identity)	*M. genitalium* essentiality^2^	Core mycoplasma genes^3^	Gene persistence in mycoplasmas^4^	Essentiality^5^ in *B. subtilis* & *E. coli*	Gene persistence^6^ in *B. subtilis* & *E. coli*
0060	tRNA modification GTPase	*trmE*	1338	0130 (56)	008 (Y)	CEMyc0050	20	N	Ec
0065	50S ribosomal protein L34	*rpmH*	150	1540 (79)	466 (Y)		20	Bs,Ec	Bs, Ec
0135	Polyamine ABC transporter permease	*potB*	837	4240 (49)	043 (Y)	CEMyc0750	20		
0140	Thiamine biosynthesis protein	*thiI*	1137	7180 (52)	372 (Y)		14		
0153*	Phosphate acetyltransferase	*pta_1*	957	2370# (55)	299 (Y)		18		
0227	Methionine adenosyltransferase	*metK*	1149	7020 (54)	047 (Y)	CEMyc01380	19	Bs,Ec	Bs, Ec
0300	Deoxyribose-phosphate aldolase	*deoC*	669	3140# (58)	050 (Y)	CEMyc0540	20		
0307	Oligosaccharide ABC transporter permease		983	0280 (43)	189 (Y)		13		
0309	Oligosaccharide ABC transporter ATP-binding protein		2103	0260 (41)	187 (Y)		13		
0311	Membrane lipoprotein P81	*mb-mp81*	2187	0240# (28)	260 (N)		8		
0317	Deoxyribonuclease IV phage-T4-induced	*nfo*	837	6210# (61)	235 (Y)	CEMyc01290	20		
0390	Glycosyltransferase		1005	7700 (32)	335.2 (Y)		7		
0464	Potassium transporter, Trk family	*ktrA*	669	1370 (51)	323 (Y)		17		
0520	Chromosome segregation protein/ABC transporter ATP-binding protein	*smc*	2979	7140# (53)	298 (N)		20	Bs	Bs
0527**	DHH family protein		1002	6920 (49)	190 (Y)		16		
0534	Hypothetical protein		456	6130 (53)	NA		17		
0629	Non-specific serine/threonine protein kinase	*pknB*	999	6850 (41)	109 (Y)		15		
0695	Drug resistance ATPase ABC transporter family, ATP-binding protein		1614	6900# (71)	NA		14		
0728	Phosphoglucomutase/phosphomannomutase domain-containing protein		2973	4840 (26)	NA		8		
0839	Chaperone protein	*dnaJ*	1134	7330 (40)	019 (Y)	CEMyc01460	12	N	Bs, Ec
0845	tRNA binding domain-containing protein		603	4860 (32)	195 (Y)		13		
0849	Methyltransferase family	*hemK*	723	1060# (53)	259 (Y)	CEMyc0140	18	N	Ec
0855	SsrA-binding protein	*smpB*	447	3520 (48)	059 (Y)	CEMyc0650	20	N	Bs, Ec

1 Essential genes in *M. pulmonis*, with percentage amino acid sequence identity with *M. bovis* strain PG45 in parentheses (French *et al.*, 2008).

# *M. pulmonis* genes found disruptable in later study (Dybvig *et al.*, 2010).

2 Gene essentiality in transposon mutagenesis studies in *M. genitalium* (Glass *et al.*, 2006).

Y indicates gene essentiality; N indicates gene dispensability; NA indicates orthologous gene not found.

3 Database of predicted essential genes http://tubic.tju.edu.cn/pdeg/ (Lin & Zhang, 2011).

4 Number of mycoplasma species (out of 20) in which gene is conserved (Liu *et al.*, 2012).

5 Essentiality in *Bacillus subtilis* (Bs) and *E. coli* (Ec). N indicates dispensability.

6 Gene persistence in *Bacillus subtilis* (Bs) and *E. coli* (Ec) (Fang *et al.*, 2005).

*0323 paralogue in *M. bovis* shares 43% identity with 0153.

**0526 paralogue in *M. bovis* shares 48% identity with 0527.

Among the other genes considered essential in earlier studies [14]–[16], [28] that were disrupted in our library were those coding for the 50S ribosomal protein L34 (RpmH, MBOVPG45_0065), the thiamine biosynthesis protein (ThiI, MBOVPG45_0140), oligosaccharide ABC transporter proteins (MBOVPG45_0307 & 0309), a glycosyltransferase (MBOVPG45_0390), the potassium transporter (KtrA, MBOVPG45_0464), a serine/threonine protein kinase (PknB, MBOVPG45_0629) and a tRNA binding domain containing protein (MBOVPG45_0845). Other genes disrupted in our study that were considered essential in the initial study on *M. pulmonis* [15], but that were found to be dispensable in a subsequent study [14], were those encoding deoxyribose-phosphate aldolase (DeoC, MBOVPG45_0300), the membrane lipoprotein P81 (Mb-mp81, MBOVPG45_0311), deoxyribonuclease IV (Nfo, MBOVPG45_0317), the chromosome segregation protein (Smc, MBOVPG45_0520), a hypothetical protein (MBOVPG45_0534), the drug resistance ABC transporter ATP-binding protein (MBOVPG45_0695), the phosphoglucomutase/phosphomannomutase domain-containing protein (MBOVPG45_0728) and the HemK methyltransferase (MBOVPG45_0849). Another two genes, encoding a phosphate acetyltransferase (MBOVPG45_0153) and a DHH family protein (MBOVPG45_0527), which were reported to be essential in earlier studies, were disrupted in the *M. bovis* library, but these genes have paralogues in the *M. bovis* genome and therefore could not be considered to be dispensable based on our study.

## Discussion

Although the genomes of the type strains of *M. bovis*, *M. agalactiae* and *M. mycoides* subspecies *mycoides* SC, all of which cause disease in ruminants, have been sequenced [7], gene essentiality data are not available for these species. There has been extensive horizontal gene transfer between these species, with many genes in *M. bovis* and *M. agalactiae* probably acquired from the phylogenetically distant *M. mycoides* cluster [8], [10] during co-infection of the same host [29]. Therefore, genes found to be non-essential in *M. bovis* are likely to also be non-essential in the other two species. Of the genes disrupted in the *M. bovis* mutant library, 48 had orthologues in *M. mycoides* subspecies *mycoides* SC and 99 had orthologues in *M. agalactiae*. Six of the 23 essential mycoplasma genes that were found to have transposon insertions in our study have essential orthologues in *B. subtilis* [30], [31].

In our study, there were insertions in 191 predicted ORFs. In earlier studies in *M. genitalium* 382 genes were found to be indispensable in *M. genitalium* [16], while 310 genes were found to be essential in *M. pulmonis* [15]. A further study in *M. pulmonis* [14] found 39 additional genes to be dispensable. Comparison of the data from our study with that obtained for *M. pulmonis* is of interest as both species have similar genome sizes and lie within the same (hominis) phylogenic group. We found 23 of the 310 genes found to be essential in the initial study on *M. pulmonis* [15] were disruptable in *M. bovis* (Table 2), with 7 of these 23 among those found to be disruptable in the later study on *M. pulmonis*.

The *M. bovis* genome has 52 ABC transporter genes, in 14 operons, and nine of these transporter gene ORFs, in four operons, were able to be disrupted (Table 3). Acquisition of nutrients by mycoplasmas appears to predominantly involve ABC transporters, and the low level of redundancy in mycoplasmas suggests that they are likely to be required for nutrient acquisition *in vivo*, but clearly some are dispensable in the complex media used for culture *in vitro*.

**Table 3:** Putative ABC transporters of *M. bovis* disrupted by transposon mutagenesis.

ORF	Product	Gene	Gene locus	Gene size (bp)	Tn insertion site in gene (%)	Operon
0116	Oligopeptide ABC transporter ATP-binding protein	*oppF2*	125479–127881	2403	72.0	0112–0116
0135*	Polyamine ABC transporter permease	*potB*	149512–150348	837	80.8	0134–0137
0307*	Oligosaccharide ABC transporter permease		344498–343515	983	39.3	0307–0311
0309*	Oligosaccharide ABC transporter ATP-binding protein		347590–345488	2103	75.8 & 76.6	0307–0311
0519	ABC transporter permease protein		596244–597584	1340	37.5	NA
0520*	ABC transporter ATP-binding protein/chromosome segregation protein	*smc*	600615–597637	2979	38.6	NA
0695*	Drug resistance ABC transporter/ATP-binding protein		797866–799479	1614	17.0	NA
0748	Glycerol ABC transporter permease	*gtsC2*	873847–873035	813	74.2	0747–0750
0749	Glycerol ABC transporter permease	*gtsB2*	874811–873837	975	25.3	0747–0750

*considered essential in earlier mycoplasma studies as described in Table 2; NA: does not appear to be part of any operon.

The dispensability of two genes belonging to the glycerol transport system, along with glycerol kinase and the glycerol uptake facilitator protein, is not surprising considering that there are two predicted glycerol transport systems in *M. bovis* that may complement each other. Earlier studies have reported that the production of hydrogen peroxide by ruminant mycoplasmas involves the glycerol transport system [32], [33], and the transport system appears to be dispensable in *M. mycoides* subspecies *mycoides* SC, as European strains, which are less virulent compared to their African counterparts, lack both the *gtsB* and *gtsC* glycerol transport genes [34].

The possibility of gene disruptions in some genes of *M. bovis* that are essential in other mycoplasmas might be expected, as there are paralogues in *M. bovis* of the genes encoding phosphate acetyltransferase and the DHH family protein. Similarly, some variations might be expected between different species because of unrecognized redundancy. The serine/threonine protein kinase gene (*pknB*), which was disrupted in the *M. bovis* library, has been reported to be essential for growth of *M. pulmonis* and *M. genitalium* [14]–[16], however *pknB* has been disrupted in *M. agalactiae* [35].

The genes MBOVPG45_0307 to MBOVPG45_0311, which are part of a putative nucleotide transporter operon [36]–[38], were disrupted in our study, and transposon insertions in *mslA*, the MBOVPG45_0311 homologue in *M. gallisepticum*, have been reported previously [39]. It has been demonstrated recently that *mslA* of *M. gallisepticum*, the MBOVPG45_0311 homologue, binds single and double stranded DNA [40], suggesting that the *mslA* may bind and deliver oligonucleotides to the exonuclease, which then processes these oligonucleotides to generate individual nucleotides for transport into the cell via the ABC transporter. The disruption of genes encoding this operon might be tolerated because there are three putative membrane nucleases in the genome of *M. bovis* strain PG45, MBOVPG45_0089, MBOVPG45_0215 and MBOVPG45_0310.

The dispensability of five genes disrupted in our study, *trmE*, *potB*, *metK*, *dnaJ* and *smpB*, which were found to be essential in all previous gene essentiality studies in mycoplasmas, and which were among the predicted set of 153 core mycoplasma genes, could not be explained by predicted redundancy. Although the polyamine transporter system is dispensable in *B. subtilis*, this may result from complementation by another transport system. It is possible that this may also be the case in *M. bovis*. The chaperone DnaJ has long been considered to be essential for cellular growth. However, as expression of DnaJ increases in response to cellular stress [41], it may be dispensable during the optimal growth conditions used for culture *in vitro*. There are no obvious explanations for the dispensability of *rpmH*, *thiI* and *ktrA*, nor for the genes encoding the glycosyltransferase and the tRNA binding domain-containing protein.

However, it has been pointed out that minimal or core sets of genes are context dependent and it has been suggested that gene persistence is a better indication of the role of specific genes in the long term survival of an organism [42] and that, in defining the minimal requirements for cellular life, it would be more useful to consider those genes that, while not ubiquitous, were conserved in most genomes. Therefore we have assessed which of the genes that we found to be dispensable (Table 2) are found in most mycoplasma genomes [43]. We have also compared the gene dispensability determined in our study with the persistence and essentiality of orthologues in *B. subtilis* and *E. coli* [44].

The dispensability of *rpm*H is surprising, as it is conserved in all the fully sequenced mycoplasma genomes [43], and not only essential in *M. pulmonis* and *M. genitalium*, but also in *B. subtilis* and *E. coli* [44]. Similarly *smc* is conserved in all the mycoplasma genomes, as well as in *B. subtilis*. In recent studies, the *rpm*H and *smc* genes have been reported to be borderline persistent [45], and *smc* could be disrupted in *M. pulmonis* [14] and *rpm*H in *B. subtilis*, although the growth of the mutant was affected [46]. The *pknB* and *thiI* genes are not highly conserved in the mollicutes, with *pkn*B absent in *M. hyorhinis*, *M. hyopneumoniae*, *M. conjunctivae* and *Acholeplasma laidlawii*, and *thi*I not found in *M. hyorhinis*, *M. hyopneumoniae*, *M. conjunctivae* or *Ureaplasma urealyticum*, its absence being correlated with a mutation in tRNAIle. The gene *hem*K, which is predicted to code for a methyltransferase, is absent in *M. conjunctivae*, *U. parvum* and *B. subtilis*, while *met*K, which codes for methionine adenosyltransferase, is conserved in all *Mycoplasma* species, *B. subtilis* and *E. coli*, but is not annotated in *U. urealyticum*.

Several potential problems with transposon-generated mutant libraries in mycoplasmas were not seen or were addressed by use of differing techniques in our study. In an earlier study [22] 16–86% of colonies growing on selective agar plates lacked a transposon insertion. In this earlier study, it was assumed that these resulted from acquisition of spontaneous resistance, but attempts to decrease the prevalence of pseudotransformants by increasing the concentration of antibiotic in selective agar failed. The problem was overcome in this earlier study by incubation of *M. bovis* in selective broth for an extended period after transformation, but this may also result in multiplication of mutants and thus increase the prevalence of replicate clones in the final library. However we did not detect any pseudotransformants following transformation with any of our transposon constructs.

Replicative transposition, resulting in multiple insertions in the genome, have been a problem in some studies. We developed several derivatives of Tn*4001*, including Tn*4001*single, which lacked one of the IS*256* arms, and minitransposons, with the transposase outside the transposon, with the aim of creating transposons that would be incapable of secondary transposition and that would thus generate mutants that could be expected to be genetically stable [47]. That this was desirable was demonstrated by the relatively high frequency of multiple insertion events we saw in mutants created using Tn*4001* (data not shown).

The potential presence of insertional hotspots has also been raised as a concern in the use of transposons to generate mutant libraries. The randomness of insertion of Tn*4001* and its derivatives was confirmed by genomic sequencing of 319 individual mutants, which demonstrated that insertion events were distributed throughout the genome (Figure 1).

Targeted gene knockout remains a challenge in mycoplasmas. Targeted gene disruption in mycoplasmas has occasionally been achieved through homologous recombination, either employing free DNA or replicable *oriC* plasmids [25], [48], [49], but the low rate of recombination has necessitated extensive passage to increase the likelihood of acquiring the desired knockout. In many cases recombination with *oriC* plasimds occurs within the *oriC* region, or in illegitimate sites, rather than in the desired targets, and if it does occur within the targeted gene it can be difficult to isolate the recombinant clone [50]. Transposon mutagenesis has been the genetic tool most commonly used to manipulate mycoplasmas because of its much greater efficiency, but there have been only limited attempts to identify mutants in libraries with specific phenotypic changes that might be attributable to disruption of specific genes. Mutant libraries have been screened for loss of reactivity with a specific antiserum against LppQ in *M. mycoides* subspecies *mycoides* SC [51], loss of gliding motility in *M. pneumoniae* [52] or loss of capacity for growth on cell cultures [35], [53]. In the absence of a selectable phenotypic trait and to avoid time consuming direct genomic sequencing of all individual mutants, the PCR based haystack mutagenesis approach [3], [51] can be used to identify specific gene knockouts. However, the approach may not be suitable for identification of gene disruptions in large coding regions, and particularly if it occurs in middle of coding regions. In our study the haystack mutagenesis approach was used to identify a *xer1* gene disruption. In earlier haystack mutagenesis studies [51], the transformants were grown in broth as a pool before DNA extraction. This may result in overgrowth of mutants with disruptions in genes not required for optimal growth. Therefore, we picked individual mutants, generated an ordered mutant library, and cultured the mutants to late log phase before creating a series of pools for screening. Instead of using two primer pairs in the Tn*4001* region [3], [51], a single oligonucleotide primer binding to the IR region of Tn*4001* was used, as it could be combined with either a forward or reverse primer flanking the gene of interest to yield a single PCR product in the event of insertion in the desired gene.

Although genome sequences are available for more than 1000 bacterial species, genome-wide essentiality data is available for only 15 species, including three *Mycoplasma* species, *M. genitalium*, *M. pneumoniae* and *M. pulmonis* [14]–[17], [28]. A set of 153 core essential mycoplasma genes have been predicted [26]. Some genes expected to be essential were identified as disrupted in an early study [28], possibly because mutants were not characterised as clonal cultures, but rather as members of a mixed pool, and some genes that were predicted to be non-essential in this initial study appeared to be essential in later studies [15].

Although the mutant library we have characterised here could not be expected to have included a comprehensive repertoire of mutatable genes as the genome was not saturated with insertions, the lack of insertions in several large genes and transport systems suggests the importance of these genes for optimal growth of *M. bovis in vitro*. These include two predicted oligopeptide ABC transporter system operons, a predicted carbohydrate uptake ABC transporter system operon and a predicted cobalt ABC transporter system operon. No gene coding for tRNAs or rRNAs, which are considered essential for cell replication, was disrupted. In addition, some large genes that encode membrane proteins or hypothetical proteins were not disrupted, including MBOVPG45_0337 (3419 bp), MBOVPG45_0481 (4547 bp) and MBOVPG45_0710 (8012 bp), and thus these genes may have a role in optimal growth of *M. bovis in vitro* and may be worthy of further investigation.

One of the largest membrane proteins in *M. bovis*, MBOVPG45_0710, which is over 8 kbp in length (2670 amino acids) and has full or partial homologues in *M. agalactiae* MAG6100, *M. fermentans* MFE_02570, *M. crocodyli* MCRO_0279, *M. synoviae* MS53_0328, *M. pulmonis* MYPU_3130, *M. conjunctivae* MCJ_003940, *M. mobile* MMOB4250, *M. hyorhinis* MYM_0289 and *M. hyopneumoniae* mhp677 [7], was not disrupted. Homologues of MBOVPG45_0710 in *M. fermentans* and *M. mobile* are predicted to possess lipase activity, and the regions between amino acid residues 90 and 395 of MBOVPG45_0710 had 31% identity to *M. hyopneumoniae* p65, which has been demonstrated to possess lipase activity [54]. Although the conserved domain is restricted to the amino terminal end of this protein, the large size and lack of disruptions within the gene suggest essentiality of this protein. It may consist of several conserved domains, the functions of which are specific for species related closely to *M. bovis*.

Thus this study has validated the use of haystack mutagenesis to identify mutants with specific genes disrupted in an ordered mutant library, and has characterised the location of more than 300 transposon insertions in the *M. bovis* genome, establishing the dispensability of at least 16 genes previously believed to be essential in mycoplasmas. These data will aid in furthering our understanding of the functions of genes and gene products of mycoplasmas.

## Methods

### Bacterial strains and culture conditions

*M. bovis* type strain PG45 (ATCC 25523) was cultured at 37°C in modified Frey's broth (21 g PPLO, 37 ml yeast extract, 100 ml inactivated swine serum, 4 ml 1.6% phenol red solution, 300 mg penicillin G, 859 ml distilled water, pH adjusted to 7.8) or on mycoplasma agar plates (modified Frey's broth without phenol red with 1% agar added). For the selection of *M. bovis* transformants, gentamicin (Invitrogen) or tetracycline (Sigma Aldrich) was added to media to a concentration of 50 µg/ml or 5 µg/ml, respectively.

*Escherichia coli* DH5α cells (Life Technologies) were used for cloning of different transposon constructs and were cultured at 37°C in Luria-Bertani (LB) broth (1% w/v tryptone (Oxoid), 0.5% w/v yeast extract (Oxoid), 0.5% w/v NaCl) with shaking at 200 rpm on an orbital shaker incubator (Ratek) or on LB agar plates (LB broth containing 1% bacteriological agar). Selection of plasmid-transformed *E. coli* DH5α cells was performed on LB agar containing 5-bromo-4-chloro-3-indolyl-β-D-galactopyranoside (X-gal) (Sigma) at 40 µg/ml, isopropyl-β-D-thiogalactopyranoside (IPTG) (Sigma) at 50 µg/ml and an appropriate antibiotic. *E. coli* DH5α containing plasmid constructs were grown in LB broth or on LB agar plates containing ampicillin (Amresco) at 100 µg/ml, gentamicin at 20 µg/ml or tetracycline at 4 µg/ml.

### Agarose gel electrophoresis and plasmid extraction

Polymerase chain reaction (PCR) products and plasmid DNA constructs were analysed using conventional agarose gel electrophoresis in 0.8–2.0% w/v agarose (Scientifix) gels in 1× TBE buffer (89 mM Tris, 89 mM boric acid, 2 mM EDTA, pH 8.0) or 0.5× TPE buffer (1× TPE is 36 mM Tris, 30 mM NaH_2_PO_4_, 1 mM EDTA) and stained with ethidium bromide at 0.1 µg/ml. DNA bands were visualised using an ultraviolet transilluminator (Gibco BRL) and imaged using either the Digital Science electrophoresis documentation and analysis system (Kodak) or the Molecular Imager ChemiDoc XRS+ imaging system (Bio-Rad).

PCR products and restriction endonuclease digestion products of plasmids were separated by agarose gel electrophoresis and the DNA in specific bands extracted using the Ultraclean gel spin DNA purification kit (Mo Bio Laboratories) according to the manufacturer's instructions. The Wizard Plus SV Minipreps DNA purification system (Promega) was used to extract up to 2 µg of plasmid DNA from *E. coli* DH5α cells, whilst for purification of 20 µg or more of plasmid DNA the Qiagen Plasmid Midi kit (Qiagen) was used according to the manufacturer's guidelines.

### Amplification of PCR products

The cleavage sites for the restriction endonucleases *Bgl*II and *Nco*I were incorporated into the forward and reverse primers, respectively, used for the amplification of Tn*4001* with either a single or both IS*256* arms. The same cleavage sites were included in the oligonucleotide primers for the amplification of the gentamicin resistance gene, while *Sac*I and *Kpn*I cleavage sites were included in the forward and reverse primers used for amplification of the transposase (*tnp*) gene (Table S1). PCR reactions were performed in a thermocycler (iCycler, Bio-Rad) with 50 pg of plasmid DNA as template in a 50 µl reaction containing 5 µl of 10× Mg^2+^ free HiFi buffer, 2 mM MgSO_4_, 250 nM of each primer, 200 µM of each deoxyribonucleotide triphosphate (dNTP) and 2.5 U of Platinum HiFi Taq DNA polymerase (Invitrogen).

### Development of novel reporter construct

To create a novel transposon from which the antibiotic resistance marker could be excised following transposon insertion in, and disruption of, a specific gene, operator and gene region fragments were designed and then synthesised commercially and cloned in the *Eco*RV site of pUC57 (GenScript Corporation). The operator region contained an inverted repeat (IR) (39 bp, 5′-gataaagtccgtataattgtgtaaaagtaaaaaggccat-3′) together with the *M. bovis tuf* promoter (252 bp *tuf* promoter region located between bases 474270 and 474521 of NCBI Reference Sequence NC_014760.1), a *vsp* signal sequence (84 bp, gene ID 10014768, predicted protein sequence MKKSKFLLLGSVASLASIPFVAAKCGET) and the *FRT* sequence (34 bp *Flp* recognition target, 5′-gaagttcctattctctagaaagtataggaacttc-3′). The gene region included the *FRT* sequence (34 bp, 5′-gaagttcctattctctagaaagtataggaacttc-3′), an *M. bovis* codon optimised alkaline phosphatase reporter gene (*phoA*) [23] and the IR (39 bp, 5′-atggcctttttacttttacacaattatacggactttatc-3′). The operator and gene segments were digested separately with *Eco*RI and *Xho*I and the operator segment ligated to the gene segment in the pUC57 backbone so that the *FRT* sequences were oriented as direct repeats. The nucleotide sequence of this novel construct, and relevant restriction endonuclease cleavage sites, are shown in Figure S1.

### Construction of plasmids carrying transposons

**Figure 2:**
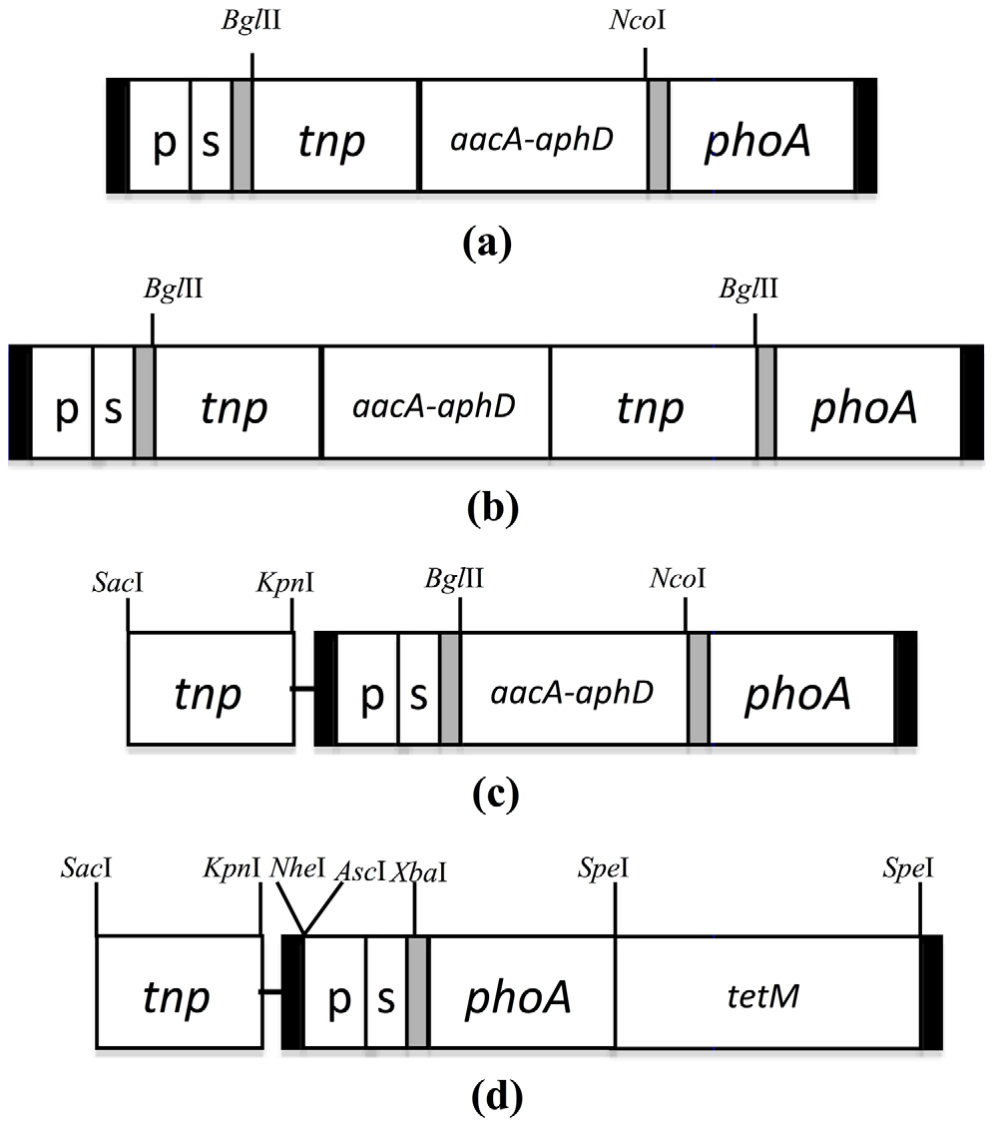
A fragment containing an inverted repeat (IR, black bar), the promoter (p), the signal sequence(s) and an *FRT* site (grey bar) was ligated to a fragment containing an *FRT* site, the reporter gene (*phoA*) and an IR using the *Eco*RI and *Xho*I cleavage sites in a pUC57 backbone. Tn*4001* with one or both insertion sequences was amplified and inserted in between the *FRT* sites of the construct to generate pTn*4001*single (a) and pTn*4001*complete (b), respectively. The construct pMiniTn*4001*-gent (c) was developed by amplifying and inserting the gentamicin resistance gene (*aacA-aphD*) between the two *FRT* sites of the construct, then the transposase gene (*tnp*) was amplified and inserted outside of the transposable element (IR, black bar). To generate the plasmid pMiniTn*4001*-tet (d), a fragment containing the IR, the promoter (p), the signal (s) and an *FRT* site was ligated to a fragment containing an *FRT* site, the reporter gene (*phoA*) and an IR in the pUC57 plasmid backbone. *FRT* sites have a unique *Xba*I cleavage site, so ligation of the fragments produced a construct with a single *FRT* site. The *tnp* gene was amplified and ligated into the plasmid outside the transposing element, then the *tetM* resistance gene with its own promoter and terminator was ligated within the construct.

Different Tn*4001*-based transposon constructs coding for gentamicin or tetracycline resistance and containing a single IS*256* arm or both IS*256* arms, and minitransposons, were generated (Figure 2). Tn*4001* containing either a single or both IS*256* arms (Figure S2), including the region coding for the gentamicin resistance gene (*aacA-aphD*), were amplified from Ptag7 [24] using the primer pairs 1SSIS256 for/2SSISgent rev and 1SSIS256 for/3SSIS256 rev, respectively (Table S1). Each PCR product was ligated to pGEM-T (Promega) and its DNA sequence confirmed by DNA sequencing using ABI PRISM Big Dye 3.1 Terminator chemistry (Life Technologies). Sequencing revealed that use of primer 1SSIS256 for had resulted in amplification of the complete Tn*4001*, resulting in inclusion of the *Bgl*II cleavage site at the 5′ and 3′ ends. Therefore, pGEM-T plasmids containing either a single IS*256* arm or the complete Tn*4001* were digested with *Bgl*II and *Nco*I or *Bgl*II alone, respectively, and ligated between the *FRT* sites of constructs digested with the same enzymes to generate the pTn*4001*single and pTn*4001*complete constructs. To facilitate insertion of the complete Tn*4001* the construct was incubated with 150 units of bacterial alkaline phosphatase (BAP, Invitrogen) at 65°C for 1 h to prevent plasmid recircularisation.

To overcome potential problems associated with subsequent transposition and multiple insertions, Tn*4001*-based minitransposons containing the genes coding for either gentamicin or tetracycline resistance were developed. For construction of pMiniTn*4001*-gent (Figure 2), the complete gentamicin resistance gene, with its promoter and terminator sequences, was amplified by PCR from the pTn*4001*single plasmid construct using the Gmgene for/Gmgene rev primer pair (Table S1), which contained engineered restriction endonuclease cleavage sites. The gentamicin resistance gene was cloned in pGEM-T, released by digestion with *Bgl*II and *Nco*I, and then ligated between the two *FRT* sites in the novel construct, which had been digested using the same pair of endonucleases. The *tnp* gene was then amplified from the pTn*4001*single plasmid using the primer pair Tnp for/Tnp rev, ligated into pGEM-T, excised with *Sac*I and *Kpn*I and then ligated into plasmid that had been cleaved with *Sac*I and *Kpn*I in a site external to the transposing element.

Another minitransposon, pMiniTn*4001*-tet (Figure 2), which had a single *FRT* site and encoded the tetracycline resistance gene (*tetM*), was also generated. In this construct, the *M. bovis* operator region was substituted with the *ltuf* promoter and *vlhA1.1* signal sequence of *M. gallisepticum* strain S6 [23]. As the *FRT* sequences contain a single *Xba*I cleavage site, ligation of the operator and gene segments after digestion with *Sac*I and *Xba*I produced a single *FRT* site (Figure S3) in the construct, with pUC57 as the backbone. The *tnp* gene was then ligated outside of the transposing element in a site exposed by digestion with *Sac*I and *Kpn*I. Finally, the *tetM* gene with its own promoter and terminator was released from pMlori [25] by digestion with *Spe*I and ligated into the *Spe*I site in the plasmid containing the *tnp* gene at the *Sac*I-*Kpn*I site.

### Transformation of *M. bovis* and creation of mutant libraries

Approximately 5 µg of each plasmid construct was used for transformation. The method used was based upon that described by Chopra-Dewasthaly *et al.* (2005), with some modifications. Briefly, 8 dilutions of a *M. bovis* culture were made in mycoplasma broth (1∶5, 1∶11.25, 1∶12.2, 1∶13.3, 1∶15, 1∶17.5, 1∶21.65 and 1∶30), and these incubated at 37°C for 16 h (late exponential phase). The cultures were pooled and cells were harvested by centrifugation at 16,000 g for 5 min at room temperature (RT) in a bench-top centrifuge. The cells were washed twice in 250 µl ice-cold HEPES–sucrose buffer (8 mM HEPES, 272 mM sucrose, pH 7.4). The cell pellet was then resuspended in 100 µl HEPES–sucrose buffer containing 5 µg plasmid DNA and transferred to a pre-chilled electroporation cuvette (0.2 cm, Bio-Rad). The mixture was kept on ice for 30 min and then pulsed (2.5 kV, 100 Ω and 25 µF) using a Gene Pulser (Bio-Rad). The cells were immediately resuspended in 1 ml cold mycoplasma broth (4°C), placed on ice for a further 15 min and then incubated at 37°C for 2 h. The transformed culture was then plated onto a selective mycoplasma plate containing 50 µg gentamicin/ml or 5 µg tetracycline/ml. The plates were allowed to dry, then incubated in the dark in an airtight canister at 37°C and examined for colonies after five days. Individual colonies were picked using a Pasteur pipette, inoculated into 500 µl broth containing an appropriate selective antibiotic, and incubated at 37°C until the colour of the medium changed. These cultures were used to create a mutant library of *M. bovis*, with each clone possessing a transposon insertion created using one of the four different constructs described above.

### PCR-based detection of the selectable marker in cloned transformants

To confirm the presence of the transposable element in the genome of the mutants, a screening PCR was performed that targeted the antibiotic resistance determinant. To verify the presence of either antibiotic resistance gene, cells from 100 µl of culture were pelleted by centrifugation at 16,000 g for 5 min at RT, the supernatant discarded and the cell pellet resuspended in 25 µl of distilled water. The resuspended cells were incubated at 100°C for 5 min and used as template for PCR. The PCR assays used 2 µl of DNA template in a 25 µl reaction mixture containing 1.25 U of Gotaq DNA polymerase (Promega) in 1× buffer supplied by the manufacturer, 200 µM of each dNTP, 1.25 mM MgCl_2_ and 250 nM of each oligonucleotide primer for amplification of the gentamicin (Gm for/Gm rev) or tetracycline (LAtetM for/LBtetM rev) resistance genes (Table S1).

### PCR-based screening for specific gene knockouts

**Figure 3:**
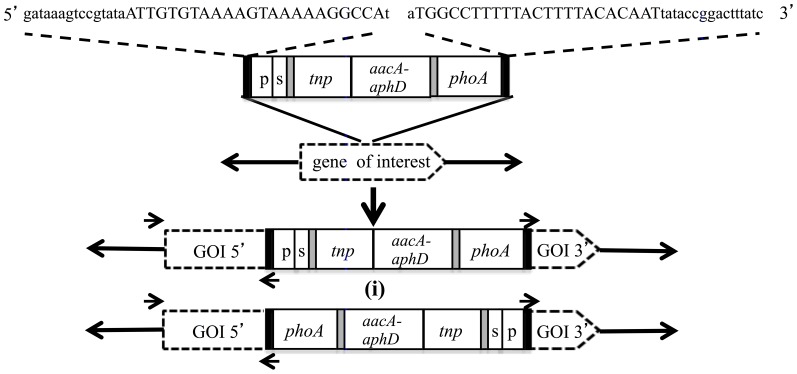
PCR-based screening approach to identify transposon insertions in gene targets. The insertion of the transposable element in a particular gene can occur in two possible orientations. PCR reaction using a primer pair, one based on the 39-bp IR sequence (uppercase) of the transposon and other one being either the forward (in this figure) or reverse primer flanking the gene of interest (GOI) would generate a single PCR product in the event of gene disruption. The relative position of the transposon insertion within the GOI is estimated based on the size of the PCR fragment including the region of binding of forward or reverse primer and primer based on IR region of transposon.

The ‘haystack mutagenesis’ approach [3] was employed to screen the library of transposon mutants for insertions in four targeted genes. To limit the number of PCR reactions, 168 individual transposon-generated mutants were cultured in 1 ml of mycoplasma broth and arranged in seven pools containing 20 to 30 mutants. The genomic DNA was extracted from these pools using the High Pure DNA purification kit (Roche). The insertion of the transposon in the genome could have occurred in either orientation (Figure 3), so the screening PCR was performed using a pairs of primers that included the IR inverse oligonucleotide, which was specific for the transposon but could bind at either end of it, and either a forward or reverse oligonucleotide specific for the gene of interest (GOI) (Table S1) to identify a pool containing the desired GOI-transposon junction. Subsequently, a similar PCR using DNA prepared by boiling a cell pellet suspended in distilled water was performed on all the individual mutants in the positive pool to identify the mutant of interest. The relative position of the transposon insertion within the GOI was estimated from the size of the PCR fragment. To confirm the location of the transposon within the specific gene, the PCR product generated was cloned into pGEM-T and its DNA sequence determined. The location of the transposon in the *xer1* gene was further confirmed by direct genome sequencing.

### Determination of transposon insertion sites in the genome

After selection from the initial agar plate each mutant was passaged a further two times in selective mycoplasma broth at 37°C to amplify the culture up to a volume of 8–10 ml. The cells were harvested by centrifugation at 11,000 g for 20 min at 4°C, washed twice in phosphate buffered saline (PBS) (140 mM NaCl, 2.7 mM KCl, 10 mM Na_2_HPO_4_, 2 mM KH_2_PO_4_), and finally resuspended in 200 µl PBS. Genomic DNA extraction was performed using the High Pure PCR kit (Roche) according to the manufacturer's protocol, except that the initial lysozyme treatment was omitted and the DNA was eluted in 50 µl of elution buffer. DNA sequencing was performed directly on genomic DNA extracted from transposon mutants. The oligonucleotide sequencing primers tuf inverse and T7 universal (Table S1), which bind within the transposon at distances of 42–67 bp and 59–78 bp, respectively, from its insertion site, were used to sequence across the transposon-genomic DNA junction. Each 20 µl reaction contained 2–3 µg of purified genomic DNA, 30 µM of the primer, 4 µl of Big Dye terminator (BDT) v3.1 enzyme mixture and 4 µl of 5× BDT dilution buffer. The sequencing products were purified and their sequence determined. The resultant DNA sequence was then used to identify the location of each transposon in the *M. bovis* PG45 genome [9] using BLAST (National Centre for Biotechnology Information, NCBI www.ncbi.nlm.nih.gov). The insertion sites were mapped onto the *M. bovis* PG45 genome using Geneious Pro 5.1.6 (Biomatters Ltd).

### Criteria for gene inactivation

To address the question of which *M. bovis* genes were dispensable for growth in laboratory media, a gene was considered to be disrupted if the transposon insertion was located after the first three codons and within the first 85% of the protein coding sequence. Global transposon disruption studies [14]–[16] have identified a repertoire of putative essential genes, and a recent study has predicted a set of 153 essential genes for all *Mycoplasma* species [26]. The dispensable genes in our *M. bovis* library were compared with the genes defined as essential in these previous studies.

## Supporting Information

Figure S1 **Nucleotide sequence of novel transposon constructs.** Relevant restriction endonuclease cleavage sites used to generate the construct are indicated above the sequence. The inverted repeat (IR) regions that act as transposable elements are marked, as well as the *tuf* promoter (p), the Vsp signal sequence (s), two directly oriented *FRT* sites and the *phoA* gene.
(TIFF)

Figure S2 **Nucleotide sequence of Tn** ***4001*** **(Ptag7) and deduced amino acid sequences of** ***tnp*** **and** ***aacA-aphD*** **.** Relevant primer binding sites are marked above the sequence, while start and stop codons of *tnp* and *aacA*-*aphD* are indicated below the sequence.
(TIFF)

Figure S3 **Nucleotide sequence of M.** ***gallisepticum*** **based transposon construct and predicted** ***phoA*** **translation.** Relevant restriction endonuclease cleavage sites are indicated above the sequence. The transposable element between the inverted repeats (IR) contains the *ltuf* promoter (p), the *vlhA1.1* signal sequence(s), a single *FRT* site and *phoA*. The predicted translation of *phoA* from the *ltuf* promoter, fused to the *vlhA1.1* signal sequence, following expected excision of the resistance marker is shown. The region outside the IRs contains the multicloning sites of the plasmid into which the region was ligated.
(TIFF)

Table S1 **Primers used for PCR in this study and their products.**
(DOCX)

Table S2 **Transposon insertions in** ***M. bovis*** **strain PG45 considered unlikely to disrupt function.**
(DOCX)

Table S3 **Transposon insertions within predicted intergenic regions in** ***M. bovis*** **strain PG45.**
(DOCX)

Table S4 **Transposon insertions within integrative conjugative elements (ICEs) in** ***M. bovis*** **strain PG45.**
(DOCX)

Table S5 **Transposon insertions within transposase genes in** ***M. bovis*** **strain PG45.**
(DOCX)
