# Supplementary figures and images for: Genes Found Essential in Other Mycoplasmas Are Dispensable in Mycoplasma bovis
Source: PLoS One. 2014 Jun 4;9(6):e97100. doi: 10.1371/journal.pone.0097100 (PMC4045577; doi:10.1371/journal.pone.0097100)

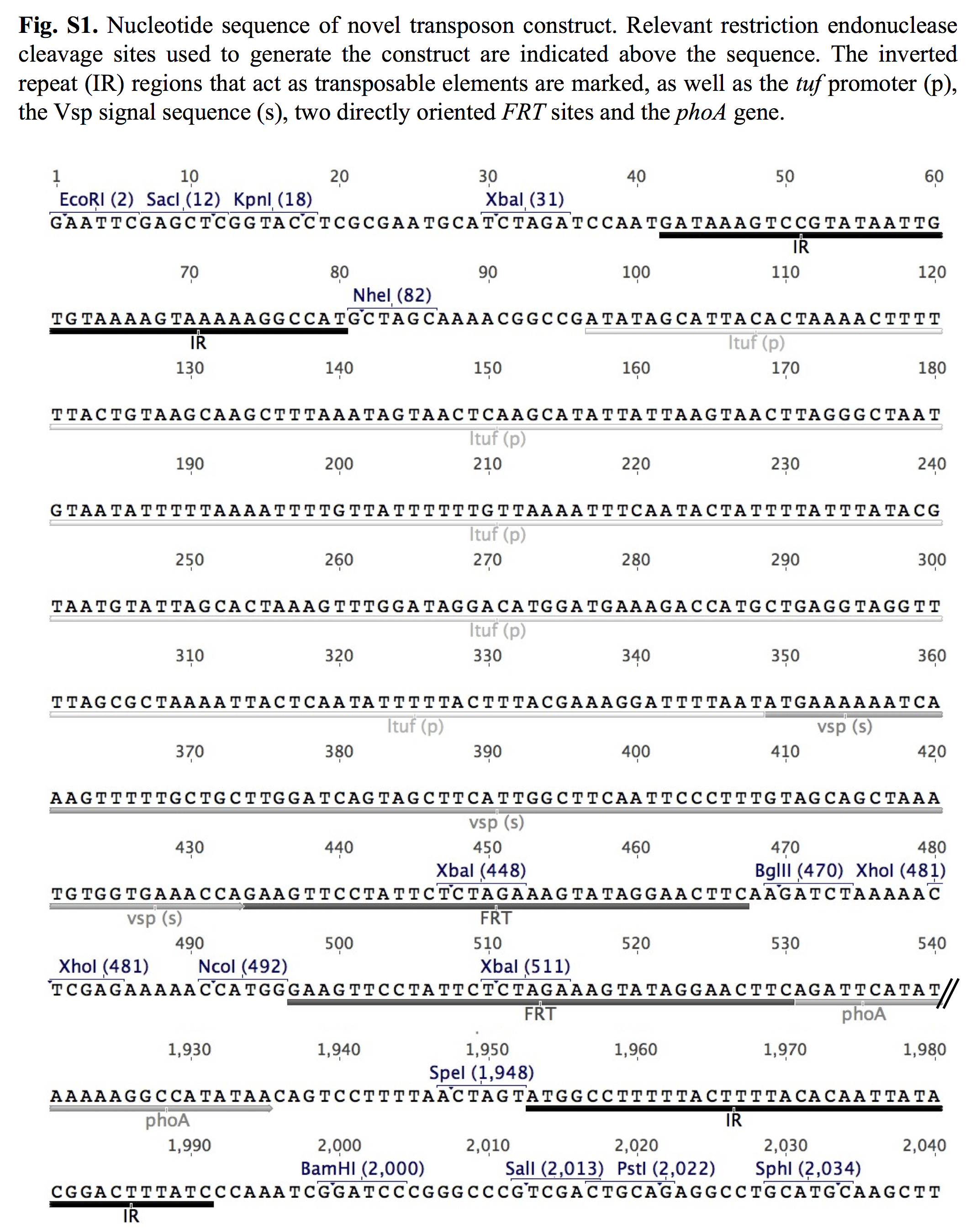

Supplement: Figure S1 — Nucleotide sequence of novel transposon constructs. Relevant restriction endonuclease cleavage sites used to generate the construct are indicated above the sequence. The inverted repeat (IR) regions that act as transposable elements are marked, as well as the tuf promoter (p), the Vsp signal sequence (s), two directly oriented FRT sites and the phoA gene. (TIFF) [file pone.0097100.s001.tiff]

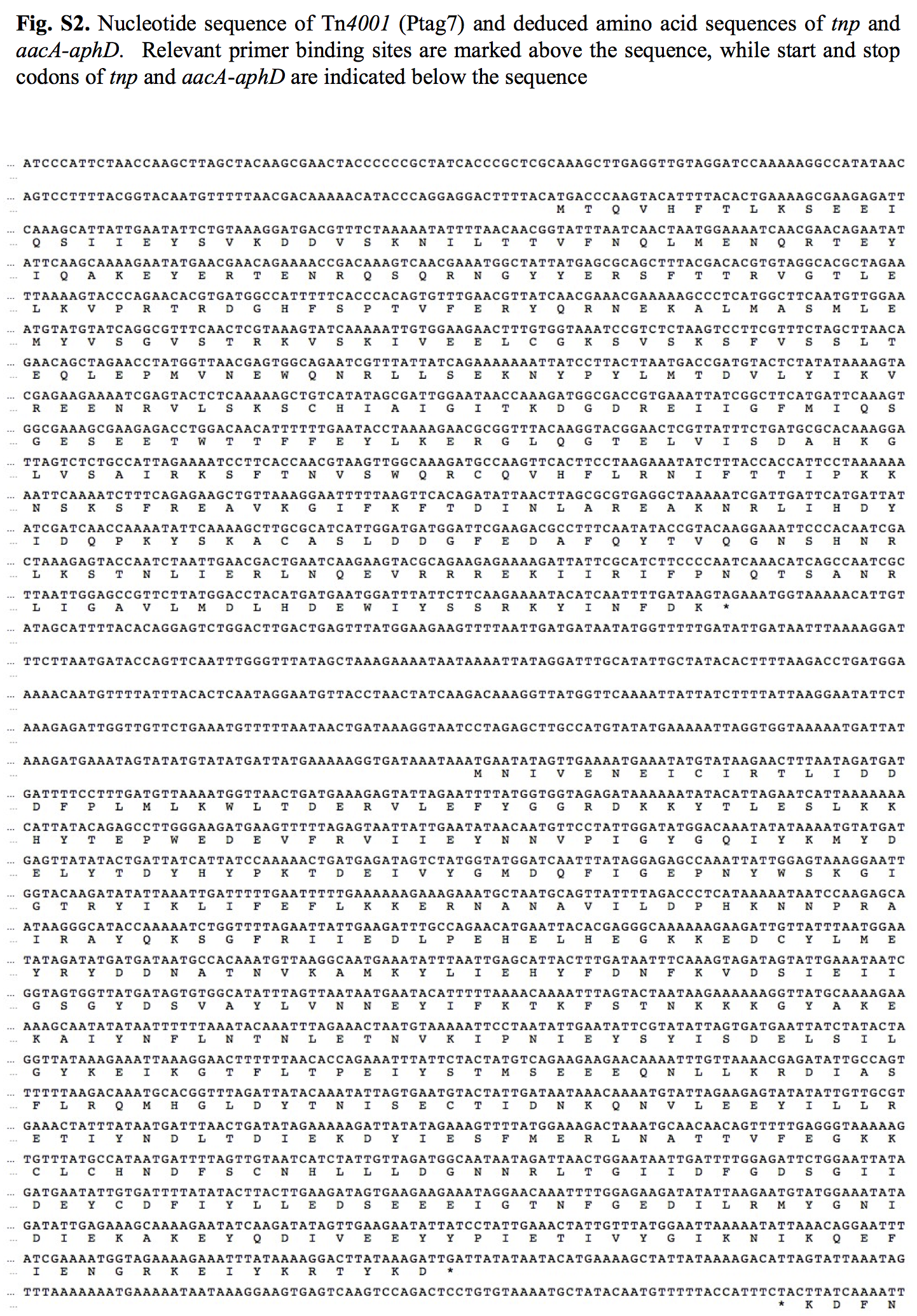

Supplement: Figure S2 — Nucleotide sequence of Tn 4001 (Ptag7) and deduced amino acid sequences of tnp and aacA-aphD . Relevant primer binding sites are marked above the sequence, while start and stop codons of tnp and aacA-aphD are indicated below the sequence. (TIFF) [file pone.0097100.s002.tiff]

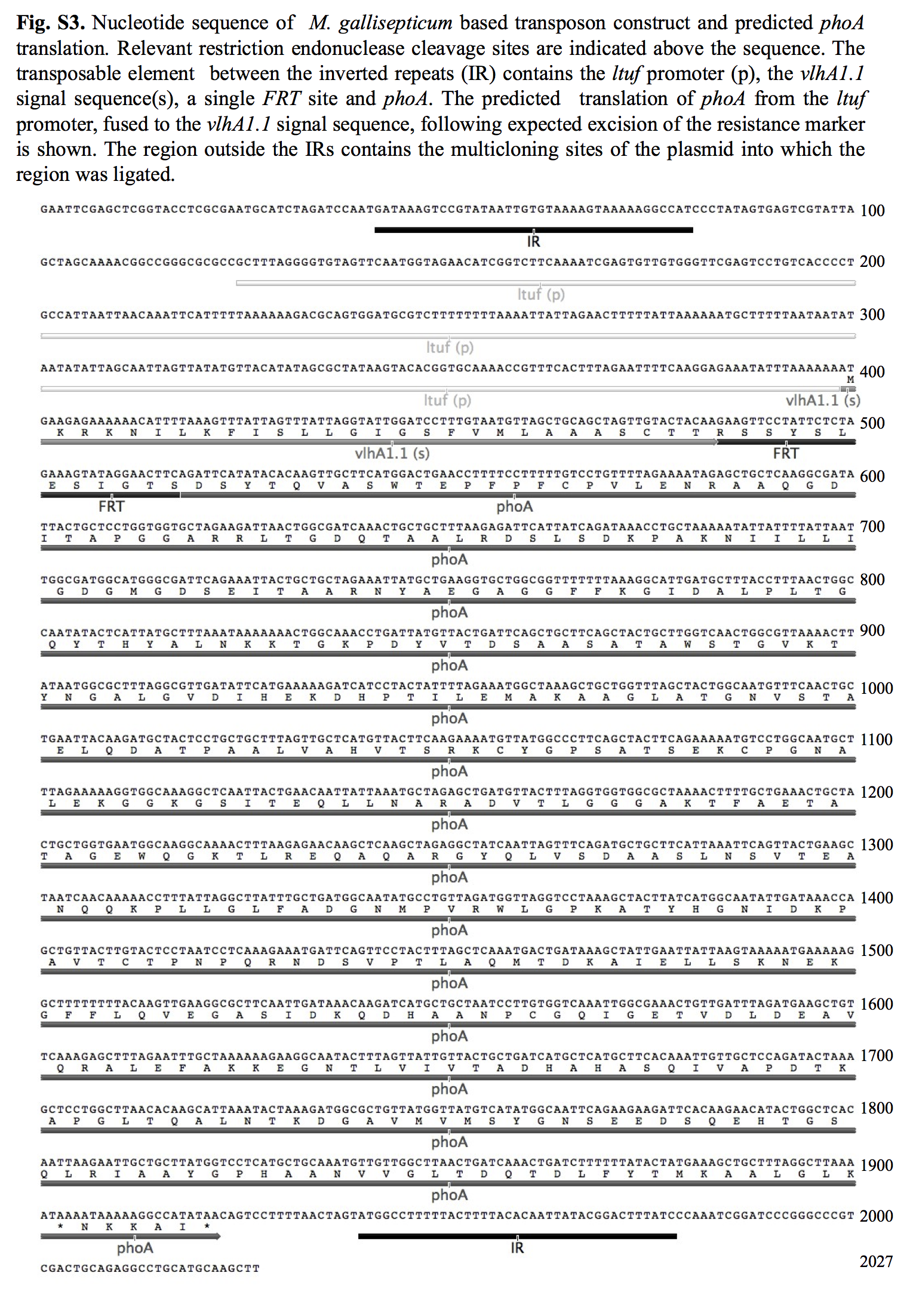

Supplement: Figure S3 — Nucleotide sequence of M. gallisepticum based transposon construct and predicted phoA translation. Relevant restriction endonuclease cleavage sites are indicated above the sequence. The transposable element between the inverted repeats (IR) contains the ltuf promoter (p), the vlhA1.1 signal sequence(s), a single FRT site and phoA. The predicted translation of phoA from the ltuf promoter, fused to the vlhA1.1 signal sequence, following expected excision of the resistance marker is shown. The region outside the IRs contains the multicloning sites of the plasmid into which the region was ligated. (TIFF) [file pone.0097100.s003.tiff]
