# Supplementary material for: Genes Found Essential in Other Mycoplasmas Are Dispensable in Mycoplasma bovis
Source: PLoS One. 2014 Jun 4;9(6):e97100. doi: 10.1371/journal.pone.0097100 (PMC4045577; doi:10.1371/journal.pone.0097100)
Supplement: Table S1 — Primers used for PCR in this study and their products. (DOCX) [file pone.0097100.s004.docx]

**Table S1.** Primers used for PCR in this study and their products

| **Primers** | **Sequence (5’-3’)** | | **PCR conditions** | | **PCR product (size)** | | **Reference** | |
| --- | --- | --- | --- | --- | --- | --- | --- | --- |
| 1SSIS256 for | | AGATCTatcccattctaaccaagc | | 94°C 5 min, 40 cycles of 94°C 30 s, 45°C 30 s, 68°C 4.5 min and 68°C 7 min | | Tn*4001* with a single IS*256* arm | | This study |
| 2SSISgent rev | | CCATGGctaatgtcttttataatagc | |  | | (3.2 kbp) | |  |
|  | |  | |  | |  | |  |
| 1SSIS256 for | | AGATCTatcccattctaaccaagc | | 94°C 5 min, 35 cycles of 94°C 30 s, 53.5°C 30 s, 68°C 4.5 min and 68°C 7 min | | Tn*4001* with both IS*256* arms | | This study |
| 3SSIS256 rev | | CCATGGaaaaaggccatataacagtc | |  | | (4.7 kbp) | |  |
|  | |  | |  | |  | |  |
| Gmgene for | | gttAGATCTgggtttatagctaaagaaaataataaaattatagg | | 94°C 5 min, 40 cycles of 94°C 30 s, 52°C 30 s, 68°C 2 min and 68°C 5 min | | Gentamicin gene | | This study |
| Gmgene rev | | gtttcaCCATGGttattatcaatctttataagtccttttataaatttc | |  | | (1.8 kbp) | |  |
|  | |  | |  | |  | |  |
| Tnp for | | GAGCTCgtgtaaaagtaaaaaggccatataacagtccttttacgg | | 94°C 5 min, 40 cycles of 94°C 30 s, 60°C 30 s, 68°C 1.5 min and 68°C 5 min | | Transposase gene | | This study |
| Tnp rev | | GGTACCttattactacttatcaaaattgatgtattttcttgaag | |  | | (1.3 kbp) | |  |
|  | |  | |  | |  | |  |
| Gm for | | ccaagagcaataagggcatac | | 95°C 2 min, 28 cycles of at 95°C 30 s, 60°C 30 s, 72°C 15 s and 72°C 5 min | | Gentamicin screening PCR & probe | | Shil, *et al.,* 2011 |
| Gm rev | | acactatcataaccactaccg | |  | | (223 bp) | |  |
|  | |  | |  | |  | |  |
| LAtetM for | | gcagttatggaagggatacg | | 94°C 3 min, 28 cycles of 94°C 45 s, 50°C 45 s, 72°C 25 s and 72°C 5 min | | Tetracycline screening PCR | | Lee *et al*., 2008 |
| LBtetM rev | | ttcttgaatacaccgagcag | |  | | (339 bp) | |  |
|  | |  | |  | |  | |  |
| GKXer1 for | | GCGGCCGCttgcagcatataaaaacatacttgc | | 94°C 5 min, 28 cycles of 94°C 30 s, 52°C 30 s, 72°C 45 s and 72°C 5 min | | Detection of *xer1* disruption | | This study |
| IR inverse | | tggcctttttacttttacacaat | |  | |  | |  |
|  | |  | |  | |  | |  |
| GKRE for | | GCGGCCGCtgttgaaacattattaccaacaaaca | | Same as above | | Detection of *type II RE* disruption | | This study |
| IR inverse | | tggcctttttacttttacacaat | |  | |  | |  |
|  | |  | |  | |  | |  |
| GKp48 for | | GCGGCCGCttgctgcttcatgtggtgat | | 94°C 5 min, 28 cycles of 94°C 30 s, 52°C 30 s, 72°C 75 s and 72°C 5 min | | Detection of *p48* disruption | | This study |
| IR inverse | | tggcctttttacttttacacaat | |  | |  | |  |
|  | |  | |  | |  | |  |
| GKoppD for | | GCGGCCGCacaataaaaagtttgcaaatccaat | | Same as above | | Detection of *oppD* disruption | | This study |
| IR inverse | | tggcctttttacttttacacaat | |  | |  | |  |
|  | |  | |  | |  | |  |
| tuf inverse | | gtaatgctatatcggccgttttgcta | | 96°C 5 min and 60 cycles of 96°C 30 s, 50°C 10 s, 60°C 4 min | | DNA sequence across the Tn-genomic DNA junction | | This study |
|  | |  | |  | |  | |  |
| T7 universal | | taatacgactcactataggg | | Same as above | | DNA sequence across the Tn-genomic DNA junction | | This study |

Upper case letters indicate the restriction endonuclease cleavage sites incorporated into the oligonucleotide primer
