# Supplementary material for: Genes Found Essential in Other Mycoplasmas Are Dispensable in Mycoplasma bovis
Source: PLoS One. 2014 Jun 4;9(6):e97100. doi: 10.1371/journal.pone.0097100 (PMC4045577; doi:10.1371/journal.pone.0097100)
Supplement: Table S2 — Transposon insertions in M. bovis strain PG45 considered unlikely to disrupt function. (DOCX) [file pone.0097100.s005.docx]

| **Table S2.** Transposon insertions in *M. bovis* strain PG45 considered unlikely to disrupt function | | | | | | | |
| --- | --- | --- | --- | --- | --- | --- | --- |
| **ORF** | **Product** | **Gene** | **Gene locus** | **Gene size (bp)** | **Proportion of gene 5’ to insertion site (%)** | **MSC orthologue^*^** | **MAGPG2 orthologue^*^** |
| 0042 | RNA methyltransferase, TrmH family, group 3 |  | 54228-54929 | 702 | 91.7 | Y | Y |
| 0122 | ABC transporter peptide-2 exporter family | Pep2E | 133401-135458 | 2058 | 94.4 |  |  |
| 0147 | Lipoprotein (PARCEL family) |  | 163466-161908 | 1558 | 98.3 |  |  |
| 0175 | Membrane protein |  | 201174-199765 | 1410 | 99.2 |  | Y |
| 0320 | Lipoprotein |  | 363023-360786 | 2238 | 86.6 88.0 | Y |  |
| 0323 | Phosphate acetyltransferase | *pta_2* | 366190-365216 | 975 | 95.4 | Y | Y |
| 0345 | Oligopeptide ABC transporter permease |  | 393935-393000 | 936 | 96.5 |  | Y |
| 0476 | Hypoxanthine phosphoribosyltransferase | *hpt* | 548888-548337 | 552 | 93.8 | Y | Y |
| 0502 | Oxidoreductase, short chain dehydrogenase/reductase family |  | 578612-577890 | 723 | 86.0 | Y | Y |
| 0543 | Oxidoreductase, zinc-binding dehydrogenase family |  | 626205-625153 | 1053 | 91.0 |  | Y |
| 0553 | RNA polymerase sigma factor | *rpoD* | 639097-640626 | 1530 | 98.9 | Y | Y |
| 0603 | LppB family lipoprotein |  | 696820-694961 | 1860 | 93.3 |  | Y |
| 0630 | Protein phosphatase 2c domain-containing protein |  | 725248-724496 | 753 | 93.4 |  | Y |
| 0634 | Ribonuclease R | *rnr* | 728104-730293 | 2190 | 98.7 | Y | Y |
| 0657 | Methyltransferase | *gidB* | 749160-749747 | 588 | 86.6 |  | Y |
| 0667 | DNA topoisomerase IV subunit A | *parC* | 759938-757353 | 2586 | 98.0 | Y | Y |
| 0686 | Membrane protein |  | 783077-784501 | 1425 | 97.4 |  | Y |
| 0751 | Membrane protein |  | 878962-876236 | 2757 | 0.2 | Y | Y |
| 0772 | Ribosomal large subunit pseudouridine synthase | *rluD* | 898539-897622 | 918 | 89.5 | Y | Y |
| 0780 | Tetrapyrrolemethylase family protein |  | 905183-904458 | 726 | 1.2 |  | Y |
| 0789 | HAD-superfamily hydrolase |  | 913566-914465 | 900 | 87.1 |  | Y |

^*^ Y indicates the presence of an orthologous gene in *M. mycoides* subspecies *mycoides* SC strain PG1 (MSC) and *M. agalactiae* strain PG2 (MAGPG2)
