# Supplementary material for: Genes Found Essential in Other Mycoplasmas Are Dispensable in Mycoplasma bovis
Source: PLoS One. 2014 Jun 4;9(6):e97100. doi: 10.1371/journal.pone.0097100 (PMC4045577; doi:10.1371/journal.pone.0097100)
Supplement: Table S3 — Transposon insertions within predicted intergenic regions in M. bovis strain PG45. (DOCX) [file pone.0097100.s006.docx]

**Table S3.** Transposon insertions within predicted intergenic regions in *M. bovis* strain PG45.

| **ORF** | **Tn insertion site** | **Intergenic region** | **5’ Flanking gene (sense)** | **3’ Flanking gene (sense)** |
| --- | --- | --- | --- | --- |
| 0040 & 0041 | 52767 | 52720-52940 | ISMbov1 (+) | *cysS* (+) |
| 0088 & 0089 | 100243 | 100230-100310 | *fba* (+) | Lipoprotein, nuclease family (+) |
| 0147 & 0148 | 163535 | 163466-163658 | PARCEL family (-) | PARCEL family (-) |
| 0148 & 0149 | 165349 | 165218-165379 | PARCEL family (-) | *ssrA* (-) |
| 0161 & 0162 | 182760 | 182612-182837 | *gidA1* (-) | Membrane protein (+) |
| 0170 & 0171 | 195365 | 195267-195395 | Type III RM system methylase (-) | *tig* (-) |
| 0173 & 0174 | 199441 | 198913-199478 | Hypothetical protein (+) | Hypothetical protein (+) |
| 0176 & 0177 | 203530 | 203450-203826 | Membrane protein (-) | Lipoprotein (+) |
| 0183 & 184* | 212399 | 212021-213200 | CDS22 (-) | CDSG (+) |
| 0887 & 0201* | 235394 | 235335-235498 | CDS13 (-) | CDS7 (-) |
| 0204 & 0205* | 240155 | 239896-240248 | Membrane protein (-) | Hypothetical protein (-) |
| 0212 & 0213* | 246326, 246526, 246768 | 246022-247227 | CDSA (-) | CDS1 (-) |
| 0234 & 0235 | 270803 | 270776-271043 | Lipoprotein (-) | Hypothetical protein (+) |
| 0243 & 0244 | 288507, 288687, 288818 | 288402-289029 | Hypothetical protein (-) | *gyrA* (+) |
| 0251 & 0252 | 297621 | 297615-297813 | Membrane protein (+) | *rplU* (+) |
| 0257 & 0258 | 301425 | 301341-301427 | Ser/Thr phosphatase family protein (-) | *recA* (-) |
| 0302 & 0303 | 339319 | 339115-339401 | Purine nucleoside phosphorylase (-) | HAD hydrolase, IIB family (+) |
| 0304 & 0305 | 341615 | 341605-341675 | GTP1/OBG family GTP-binding protein (+) | Lipoprotein (-) |
| 0311 & 0312 | 351058 | 351020-351129 | *mb-mp81* (-) | ISMbov4 (-) |
| 0319 & 0320 | 360650 | 360486-360786 | Membrane protein (-) | Lipoprotein (-) |
| 0340 & 0341 | 387720 | 387476-388014 | *infC* (-) | Lipoprotein (+) |
| 0415 & 0416 | 479240 | 479218-479241 | peptidase M22 family (+) | Lipoprotein (+) |
| 0425 & 0426 | 495075, 495701 | 495066-495979 | Membrane protein (-) | Hypothetical protein (-) |
| 0432 & 0433 | 502921 | 502855-503133 | ISMbov4 (+) | Membrane protein (-) |
| 0496 & 0497 | 572185 | 572181-572944 | Hypothetical protein (-) | ISMbov5 (-) |
| 0499 & 0500 | 576619 | 576525-576720 | ISMbov3 (+) | Hypothetical protein (-) |
| 0501 & 0502 | 577582 | 577565-577890 | Hypothetical protein (-) | Oxidoreductase, short chain dehydrogenase/reductase family (-) |
| 0614 & 0615 | 703330 | 703057-703535 | tRNA-Pro (-) | Type I RM system R subunit (+) |
| 0681 & 0682 | 778090 | 778027-778142 | Membrane protein (+) | Lipoprotein, LppA (-) |
| 0684 & 0685 | 780695 | 780612-780789 | Conserved hypothetical protein (+) | Membrane protein (+) |
| 0686 & 0687 | 784595 | 784501-785008 | Membrane protein (+) | *dnaE* (+) |
| 0697 & 0698 | 802141 | 802109-802376 | Lipoprotein (-) | ISMbov5 (-) |
| 0714 & 0715 | 832426 | 832404-832431 | *rplL* (-) | *rplJ* (-) |
| 0739 & 0740 | 861760 | 861740-861975 | Hypothetical protein (+) | Lipoprotein (-) |
| 0795 & 0796 | 918400 | 918392-918533 | Membrane protein (-) | *apt* (-) |
| 0810 & 0811 | 935725 | 935674-935859 | *vspG* (-) | *vspN* (-) |
| 0820 & 0821 | 947561 | 947475-947734 | *vspI* (+) | *vspE* (-) |
| 0822 & 0986 | 949240 | 949161-949320 | *xer1* (+) | tRNA-Lys (+) |
| 0835 & 0836 | 962354 | 962212-962386 | Holo-acyl carrier protein synthase (-) | *engA* (-) |

*part of ICEB2; + gene coded on same strand as disrupted strand; - gene coded on strand complementary to disrupted gene
