# Supplementary material for: Genes Found Essential in Other Mycoplasmas Are Dispensable in Mycoplasma bovis
Source: PLoS One. 2014 Jun 4;9(6):e97100. doi: 10.1371/journal.pone.0097100 (PMC4045577; doi:10.1371/journal.pone.0097100)
Supplement: Table S4 — Transposon insertions within integrative conjugative elements (ICEs) in M. bovis strain PG45. (DOCX) [file pone.0097100.s007.docx]

| **Table S4.** Transposon insertions within integrative conjugative elements (ICEs) in *M. bovis* strain PG45. | | | | |
| --- | --- | --- | --- | --- |
| **ORF*** | **Gene/ Product** | **Gene locus** | **Gene size (bp)** | **Proportion of gene 5’ to insertion site (%)** |
|  | **ICEB2** |  |  |  |
| 0187 | CDS14 | 217679-216111 | 1569 | 43.3, 78.6 & 97.1 |
| 0189 | Conserved hypothetical protein | 220236-218521 | 1716 | 49.7 |
| 0192 | ISMbov1 | 223514-222264 | 1251 | 16.3 |
| 0193 | ISMbov2 | 223517-225189 | 1413 | 30.9 |
| 0194 | CDS19 | 228629-225216 | 3414 | 43.6 & 99.5 |
| 0195 | CDS17 | 229876-228635 | 1242 | 65.2 |
| 0198 | CDS17 | 233508-233062 | 1395 | 41.7 |
| 0201 | CDS7 | 236487-235498 | 990 | 46.6 |
| 0202 | CDS5 | 238548-236536 | 2013 | 17.5, 21.5, 21.9, 23.5, 24.9, 57.7 & 98.9 |
| 0207 | Conserved hypothetical protein | 242769-242110 | 660 | 25.6 |
| 0208 | Conserved hypothetical protein | 243420-242785 | 636 | 0.8 |
| 0209 | CDSB | 243867-243436 | 432 | 23.2 |
| 0210 | CDS11 | 244562-243891 | 672 | 74.0 |
| 0211 | CDS12 | 245003-244575 | 429 | 48.0 |
| 0212 | CDSA | 246022-245009 | 1014 | 2.2 & 93.7 |
| 0213 | CDS1 | 248018-247227 | 792 | 39.5 |
|  |  |  |  |  |
|  | **ICEB1** |  |  |  |
| 0481 | Membrane protein | 557194-552647 | 4548 | 18.8 & 77.7 |
| 0483 | *traE* family protein | 560653-557828 | 2583 | 11.8 |
| 0487 | Lipoprotein | 565238-563730 | 1509 | 43.6 |
| 0489 | Hypothetical protein | 566802-566140 | 663 | 5.6 & 35.0 |
| 0492 | Hypothetical protein | 568334-567483 | 852 | 82.6 |
| 0495 | Hypothetical protein | 572022-570661 | 1395 | 43.2 |
| * six additional insertions were in intergenic regions within ICEB2 as shown in Table 4 | | | | |
