# Supplementary material for: Genes Found Essential in Other Mycoplasmas Are Dispensable in Mycoplasma bovis
Source: PLoS One. 2014 Jun 4;9(6):e97100. doi: 10.1371/journal.pone.0097100 (PMC4045577; doi:10.1371/journal.pone.0097100)
Supplement: Table S5 — Transposon insertions within transposase genes in M. bovis strain PG45. (DOCX) [file pone.0097100.s008.docx]

| **Table S5.** Transposon insertions within transposase genes in *M. bovis* strain PG45 | | | | | |
| --- | --- | --- | --- | --- | --- |
| **ORF** | **Transposase Product** | **Number of insertions** | **Gene locus** | **Gene size** | **Proportion of gene 5’ to insertion site (%)** |
| 0040 | ISMbov1 | 2 | 51470-52720 | 1250 | 46.4 & 89.6 |
| 0166 |  | 2 | 186160-187410 | 1250 | 4.1 & 20.8 |
| 0661 |  | 1 | 752108-750858 | 1250 | 94.1 |
| 0676 |  | 1 | 771312-770062 | 1250 | 54.6 |
| 0786 |  | 3 | 910976- 909726 | 1250 | 25.8, 88.6 & 90.1 |
| 0804 |  | 1 | 927369-928526 | 1157 | 14.8 |
| 0014 | ISMbov2 | 1 | 12164-10492 | 1672 | 99.5 |
| 0181 |  | 1 | 210111-208700 | 1411 | 60.5 |
| 0571 |  | 1 | 665485-664216 | 1269 | 12.8 |
| 0499 | ISMbov3 | 1 | 574851-576524 | 1673 | 59.2 |
| 0842 |  | 1 | 967055-968728 | 1673 | 26.9 |
| 0143 | ISMbov4 | 4 | 156740-158505 | 1765 | 7.4, 20.8, 38.2 & 69.6 |
| 0432 |  | 6 | 501422-502855 | 1433 | 11.7, 16.3, 27.4, 36.6, 86.4 & 93.1 |
| 0625 |  | 1 | 718739-717306 | 1433 | 14.9 |
| 0497 truncated | ISMbov5 | 1 | 572946-573215 | 269 | 68.4 |
| 0698 |  | 2 | 802593-803981 | 1388 | 31.1 & 76.4 |
| 0217 | ISMbov6 | 5 | 252950-251934 | 1016 | 40.4, 51.3, 65.0, 69.7 & 75.6 |
| 0429 |  | 1 | 498522-499538 | 1016 | 6.2 |
| 0707 |  | 1 | 811770-813168 | 1398 | 1.4 |
| 0817 |  | 1 | 942195-943211 | 1016 | 48.7 |
| 0870 |  | 1 | 23258-23410 | 152 | 70.4 |
| 0242 | ISMbov7 | 1 | 283594 - 282350 | 1244 | 24.3 |
| 0627 |  | 2 | 721177-721922 | 755 | 83.2 & 98.7 |
| 0708 |  | 1 | 814312-813050 | 1262 | 4.6 |
| 0745 |  | 2 | 868597-869840 | 1243 | 15.1 & 38.1 |
| 0857 |  | 4 | 979385-980637 | 1252 | 28.8, 49.1, 55.1 & 92.6 |
| 0861 |  | 2 | 983855-985099 | 1244 | 14.5 & 29.7 |
